# An Adaptive Spreading Factor Selection Scheme for a Single Channel LoRa Modem

**DOI:** 10.3390/s20041008

**Published:** 2020-02-13

**Authors:** Seungku Kim, Heonkook Lee, Sungho Jeon

**Affiliations:** 1 School of Electronics Engineering, Chungbuk National University, Cheongju 28644, Korea; satosily@gmail.com; 2 ESS R&D Center, LG Chem Ltd., Daejeon 34122, Korea; datafort@naver.com

**Keywords:** LoRa, LoRaWAN, LPWAN, multi-hop, spreading factor

## Abstract

When the low power wide area network (LPWAN) was developed for the internet of things (IoT), it attracted significant attention. LoRa, which is one of the LPWAN technologies, provides low-power and long-range wireless communication using a frequency band under 1 GHz. A long-range wide area network (LoRaWAN) provides a simple star topology network that is not scalable; it supports multi-data rates by adjusting the spreading factor, code rate, and bandwidth. This paper proposes an adaptive spreading factor selection scheme for corresponding spreading factors (SFs) between a transmitter and receiver. The scheme enables the maximum throughput and minimum network cost, using cheap single channel LoRa modules. It provides iterative SF inspection and an SF selection algorithm that allows each link to communicate at independent data rates. We implemented a multi-hop LoRa network and evaluated the performance of experiments in various network topologies. The adaptive spreading factor selection (ASFS) scheme showed outstanding end-to-end throughput, peaking at three times the performance of standalone modems. We expect the ASFS scheme will be a suitable technology for applications requiring high throughput on a multi-hop network.

## 1. Introduction

Within the internet of things (IoT), objects connect to the internet and provide useful services by exchanging information. The types of service impact the communication technology requirements: data rate, communication range, energy efficiency, delay, and security. At the inception of IoT, small-scale networks used short-range wireless communication technologies such as WiFi, ZigBee, and Bluetooth; however, the diversification of IoT services increases the need for large-scale networks. Although using mobile communication such as LTE increases the scale, the high cost and high energy consumption negate the benefit. To implement large-scale IoT networks without the disadvantages, providers increasingly choose between low power wide area network (LPWAN) technologies: narrowband internet of things (NB-IoT), long term evolution (LTE), category M (CAT-M), Sigfox, and long range (LoRa).

LoRa Alliance released the LoRaWAN specification in 2015, and Semtech developed LoRa chipset. LoRa uses the industrial scientific medical (ISM) frequency bands for Europe and North America: 868 and 915 MHz, respectively. The maximum data rate is 5.47 kbps, and the maximum communication range is 2 km in non-line of sight (NLOS) conditions and 20 km in line-of-sight (LOS) conditions [1]. The modulation in LoRa uses chirp spread spectrum (CSS) technology and the corresponding linear wideband modulated chirp pulses. Within CSS, each bit of the message is encoded as multiple chirps; each chirp is a sinusoidal signal whose frequency increases or decreases linearly over time. Because the CSS technology uses wideband technology to transmit signals, it tolerates noise and multipath fading, and because the chirp functions regardless of the signal attenuation, CSS resists the Doppler effect.

LoRa is a CSS based physical layer, and LoRaWAN [2] is a data link layer based on LoRa, which provides a star topology network between a gateway and end-devices. An end-device known as a mote consists of sensors or actuators; it has one of three class types: A, B, or C, depending on the application requirements. The LoRaWAN gateway, known as a concentrator, relays messages between end-devices and a network server via the Internet.

The spreading factor (SF) impacts the communication performance of LoRa, which uses an SF between 7 and 12. A larger SF increases the time on air, which increases energy consumption, reduces the data rate, and improves communication range. For successful communication, as determined by the SF, the modulation method must correspond between a transmitter and a receiver for a given packet.

The LoRaWAN gateway (e.g., SX1301 [3]) communicates over multi-channels with multi-spreading factors. With this technique, end devices simultaneously communicate with the gateway using different channels and data rates without pre-negotiation and enabling the gateway to accommodate about 10,000 end-devices at the same time. However, in the multi-data rate channel mode, the gateway is limited to a 125 kHz bandwidth with eight channels, even if the maximum bandwidth of LoRa is 500 kHz.

The LoRaWAN constructs single-hop networks with a high-cost/high-performance gateway and low-cost/low-performance end-devices. Although this asymmetric structure allows a simple and low-cost LoRaWAN, the single-hop network limits operational attributes [4], such as scalability. Therefore, we also consider a multi-hop LoRa network for applications requiring an extensive network because the asymmetric structure of the LoRaWAN is not suitable for constructing multi-hop networks: High-performance gateway routers increase network deployment costs. The maximum throughput is also low because the entire network bandwidth is limited to 125 kHz for multi-channel support. Finally, when a low-performance end-device is used as a router, the data rate of the entire network is limited to that of the end device with the highest SF.

In this paper, we propose an adaptive spreading factor selection (ASFS) scheme that enables every device, supporting a single-data rate to achieve a multi-data rate. The ASFS allows a transmitter and a receiver to synchronize their SFs without any packet exchanges. We implemented a multi-hop LoRa network and the ASFS scheme on real LoRa devices. The evaluation results indicate that the ASFS scheme improves the end-to-end throughput and network throughput 

The rest of this paper is organized as follows. Section 2 briefly introduces the background, and Section 3 describes the related works. In Section 4, we propose an adaptive spreading factor selection scheme for multi-hop LoRa networks. In Section 5, we present the experimental results and the performance evaluation, and the conclusions are drawn in Section 6.

## 2. Background

In this section, we describe the background of LoRa and LoRaWAN.

### 2.1. LoRa

LoRa, an LPWAN technology, uses CSS modulation at frequencies below 1 GHz. CSS modulates the data symbols into chirp signals whose frequency continually changes over time. The LoRa frame starts with along preamble chirp, helping the receiver in LoRa signal detection. Because the preamble of LoRa is the same for each transmitter, the end of the preamble is separated by two sync words [1].

The parameters used by LoRa are SF, coding rate (CR), and bandwidth (BW). SF is given by

(1) $$SF=log_{2}\left(R_{c}/R_{s}\right)$$

where Rc and Rs are chip rate and symbol rate, respectively [5]. SF denotes the number of chips per symbol and ranges between 7 and 12. For LoRa, the data rate parameter is determined as follows:

(2) $$Data\;rate=SF\times \frac{BW}{2^{SF}}\times CR$$

SF determines the number of chips per symbol, which is inversely proportional to the modulation rate of the chirp. Ranging between 4/5 and 4/8, CR is the ratio of non-redundant data to all data within the transmit and receive frames. BW can take one of three values: 125, 250, or 500 kHz. Because they affect LoRa packet modulation and demodulation, these parameters must agree between transmitter and receiver for successful communication. The symbol rate is given by the following:

(3) $$R_{s}=\frac{BW}{2^{SF}}$$

The chirp rate can be derived by the symbol rate of (3).

(4) $$Chirp\;rate=BW\times R_{s}=\frac{BW^{2}}{2^{SF}}$$

If the BW of LoRa is constant, the chirp rate differs according to the SF. For each SF, the orthogonality of the chirp prevents interference with any other [6]; when two or more transmitters use the same channel to simultaneously transmit with different SFs, the packets do not interfere with each other [7].

The receive sensitivity parameter for LoRa is defined below:

(5) Sensitivity=−174+10log(BW)+NF+SNR

where NF is the noise floor that is fixed for a given hardware, and, inversely proportional to SF, SNR is the signal to noise ratio [2]. Therefore, as BW decreases and SF increases, the sensitivity decreases, enabling the extension of communication distance.

LoRa provides a channel activity detection (CAD) mode, which quickly detects the LoRa preamble on the channel. The receiver compares the collected preamble samples from the CAD to the ideal preamble samples. Once the comparison process completes, the CadDone interrupt occurs, and the system returns to standby mode. If preamble and ideal are matched, a CadDetected interrupt is set. The required time for CAD is given as follows [8]:

(6) $$T_{CAD}=\frac{2^{SF}+32}{BW}$$

CAD time is proportional to SF and inversely proportional to BW. Because CAD depends on the LoRa modulation, the receiver can only detect the preamble transmitted with the same SF and BW.

### 2.2. LoRaWAN

LoRaWAN specifies a star topology-based MAC protocol over the LoRa physical layer. It defines three device types: Class A, Class B, and Class C, according to the application requirements. The LoRaWAN consists of a gateway and multiple end-devices. To save battery life, a single-hop with simple protocols connects the gateway and end-devices. The gateway allocates the SF, transmission power, and the channel allocated for end-device communication. It typically uses a dedicated module (e.g., SX1301 [3]) supporting multi-channel and multi-data rates. It simultaneously demodulates up to eight LoRa packets with random spreading factors on random channels. 

An adaptive data rate (ADR) in LoRaWAN optimizes data rate, airtime, and energy consumption. If the LoRaWAN uses the ADR, the gateway divides the frequency band into eight 125 kHz channels and listens for the uplink frame in any channel and data rate. In each channel, the LoRaWAN network server calculates the margin of SNR for each SF and determines a proper SF and transmission power for the static end-devices; the data rates range from 0.3 kbps to 50 kbps.

When using a gateway that supports multi-channel and multi-data rate, LoRaWAN can accommodate a massive number of end-devices. In indoor environments with many obstacles, the communication coverage decreases; hence, it is difficult to expect high-density end-devices in the network. In addition, the single-hop star topology of LoRaWAN restricts the network scalability: if a new end-device exceeds the gateway communication radius, a new network with an additional gateway should be built for it. Some studies [4,9] have already addressed the need for multi-hop LoRa networks.

## 3. Related Works

In this section, we introduce existing research related to multi-hop LoRa network construction and SF control. M. Bor et al. [6] proposed LoRaBlink; it supports multi-hop LoRa networks over the LoRa’s physical layer. The data link layer uses beacons to implement time-division multiplexing, enabling network-layer support for the flooding algorithm. They built a multi-hop network containing six LoRa devices and evaluated its performance. C. Liao et al. [9] present CT-LoRa, a concurrent transmission (CT) protocol for multi-hop LoRa networks. Through synchronized packet collisions, CT enhances network reliability. They use flooding for the message routing, which achieves fast packet broadcast through CT. Their results showed high reliability in multi-hop networks. G. Zhu et al. [10] proposed combining an SF-Pipeline with CT [9] to construct multi-hop LoRa networks. The SF-Pipeline reduces SF as the number of hops transmitted via the CT increases. Their evaluation results indicate that SF-Pipeline improves the reliability of CT-LoRa.

B. Reynders et al. [11] propose a solution for the near-far effect in LoRaWAN. The near-far effect means that a packet of an end-device far from the gateway suffers destructive collisions from a packet of an end-device close to the gateway. They propose an algorithm to optimization SF and transmission power and validate the decrease of packet error rate via simulation. D. Zorbas et al. [12] present combinations of bandwidths and spreading factors to enhance the capacity of LoRa networks and compute the packet success ratio in each combination. Using these computations, a gateway maximizes the network capacity by optimizing the SF distribution to end-devices. D. Croce et al. [13] evaluate collisions in high-density LoRa networks. As SFs differed, they both analyzed the packet loss probability and, via commercial LoRa devices and software-defined values, proved imperfect orthogonality. Moreover, when they used large SFs in congested networks, packets were more susceptible to collisions. F. Cuomo et al. [14] propose EXPLoRa to allocate suitable SFs to end-devices. EXPLoRa selects the SF based on distance, received signal strength indicator (RSSI), and the number of connected end-devices in the network. The ordered waterfilling approach in EXPLoRa allocates SFs to maintain the same airtime for all end-devices. In high-density networks, it significantly improved data rate and robustness compared to the ADR mechanism.

## 4. An Adaptive Spreading Factor Selection Scheme

In LoRaWAN, using special hardware [3], ADR enables multi-channels and multi-data rates in a gateway. ADR is an economical and efficient way to build a single-hop network; however, when constructing multi-hop networks, we need to choose between using routers as gateway hardware (e.g., SX1301) and using routers as end-device hardware. The gateway is more expensive than the end-device but enables the use of ADR with the connected end-devices. If we use routers as the gateway hardware, the cost of the network increases, the router supports multi-channel and data rates, and the limited 125 kHz bandwidth for the ADR [3] reduces the interference effect but degrades the maximum data rate of each link. Alternatively, if we use routers as the end-device hardware, the cost of the network decreases, the router only supports a single channel and data rate, and the channel and data rate are fixed in the uplink and downlink of each router, which limits the data rate of the entire network to the lowest among all routers.

We exploit routers as single-channel end-device hardware for cost-effective and high data rate multi-hop LoRa networks. Additionally, we consider how to optimize the data rate per link because ADR is unavailable in single-channel end-device hardware. In this paper, we propose an innovative adaptive spreading factor selection (ASFS) scheme for the multi-hop LoRa network. During the preamble sampling period, the receiver estimates the proper SF of the transmitter. Through this estimation, the receiver synchronizes the SF with the transmitter without any packet exchanges. ASFS benefits the routers of multi-hop LoRa networks that retain multiple links.

**Figure 1:**
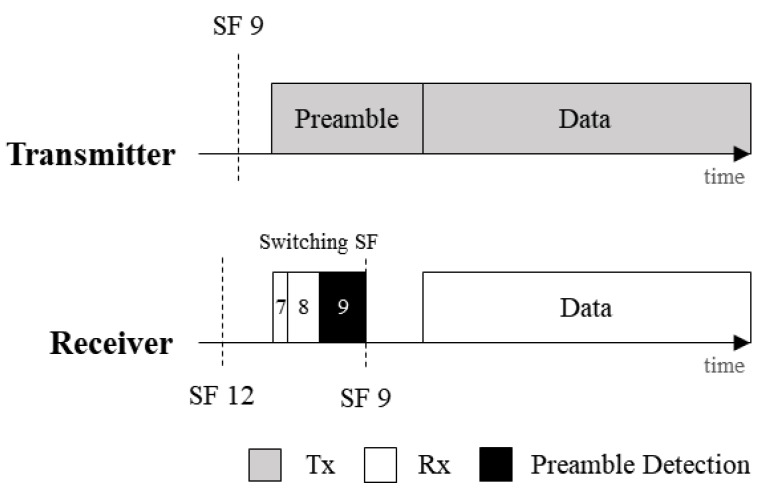
An example of the ASFS operation.

To check the presence of LoRa signals, channel activity detection (CAD) is performed. The CAD mode efficiently detects the LoRa preamble signal [7,8]. A receiver in CAD mode only detects a preamble with a matching SF. In the ASFS, the receiver inspects the preamble by sequentially changing its SF from the lowest value (SF7) to the highest value (SF12). When a receiver detects a preamble in a specific SF, the receiver learns the corresponding SF of a transmitted packet. As shown in Figure 1, the receiver uses CAD mode to find a proper SF during the preamble period by switching its SF while the transmitter sends a packet with SF9. After this process, the receiver synchronizes the SF with the transmitter and processes the data.

**Figure 2:**
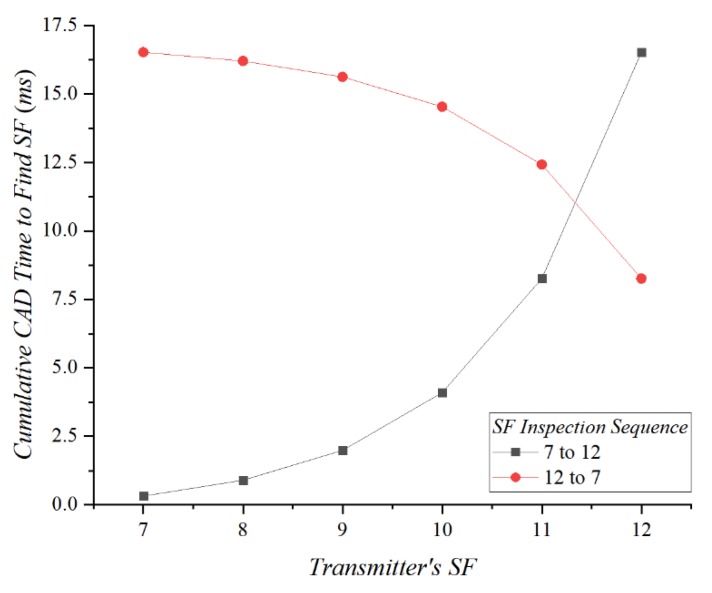
CAD time for ascending and descending selection sequences.

In ASFS, the number of steps in the SF inspection sequence determines the preamble sampling time for finding a proper SF. As in Equation (6), if BW is constant, the CAD time exponentially increases according to the SF value. Figure 2 shows the theoretical cumulative CAD time needed to find the SF of the transmitter by using the SF inspection sequence. If the receiver examines the preamble in ascending order from SF7 to SF12, the average cumulative CAD time is 5.35 ms; the lower SF consumes less CAD time. Alternatively, when the receiver inspects the preamble in descending order from SF12 to SF7, the average cumulative CAD time is 13.92 ms. Because the average cumulative CAD for the descending SF inspection order is more than two times greater, we propose searching SFs in ascending order.

During the ASFS sequence, the preamble sent by the transmitter shall be long enough for the receiver to find a proper SF during the CAD mode. The preamble duration is given by [5]:

(7) $$T_{preamble}=\left(n_{preamble}+4.25\right)\times T_{symbol},$$

where *n_preamble_* and *T_symbol_* are the number of preamble symbols and the symbol period, respectively. The *n_preamble_* ranges from 6 to 65535. *T_symbol_* is 1/*R_s_* where *R_s_* is obtained by Equation (3). We calculated *T_preamble_* and cumulative *T_CAD_* as in Table 1, where BW is 500 kHz, and *n_preamble_* is 6. Table 1 indicates that the receiver has enough time to find a proper SF even if the transmitter sends the preamble with minimum length.

**Figure 3:**
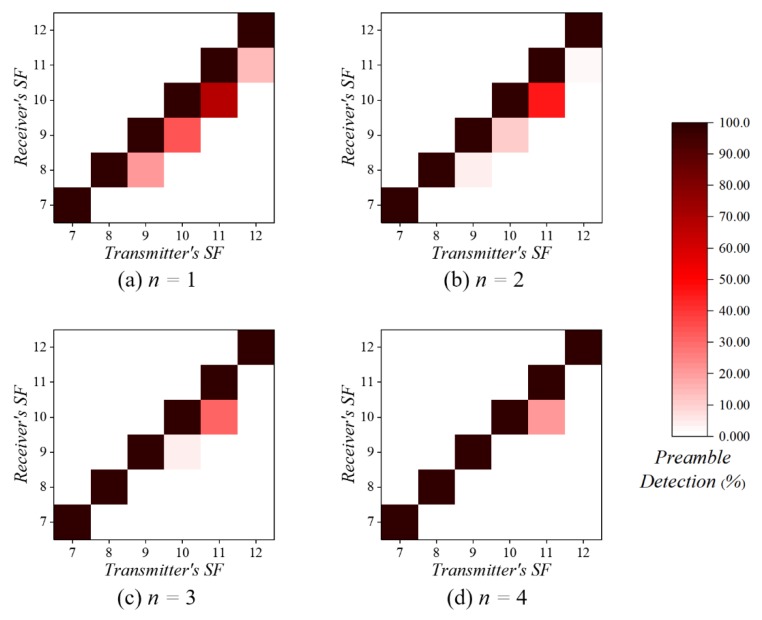
The probability of preamble detection according to the number of spreading factor (SF) inspection repetitions.

As described in Section 2. A, each SF is theoretically orthogonal; in LoRa, concurrent transmissions with different SFs do not interfere with each other. Figure 3 shows the probability of preamble detection in different transmitter and receiver SF combinations. For this experiment, the transmitter repeatedly sends 100,000 packets at fixed intervals with the predefined SFs, and the receiver detects the preamble while sequentially changing the SF every interval. The preamble is always detected if the SF of the transmitter and the receiver match; however, the preamble of adjacent SFs is sometimes detected in all SFs. Therefore, in practice, LoRa has imperfect SF orthogonality like [13].

The quasi-orthogonality of the SFs may cause the receiver to detect the wrong preamble (false SF selection). For example, the receiver may incorrectly detect the preamble transmitted in SF10 as SF9 or SF11. Although not well distinguished in the figure, the receiver detects preambles sent with a lower SF less than 1% of the time. This disrupts packet reception.

We propose two solutions: an iterative SF inspection and a modified SF selection algorithm. In iterative SF inspection, the receiver repeats the SF inspection during preamble sampling. The receiver selects the SF that is detected by all repetitions. A probability that the preamble of a specific SF is detected in all repetitions is given by the following:

(8) $$P_{SF_{i}}=P_{detect}{}^{n}$$

where *P_detect_* is a preamble detection probability at a specific SF, and *n* is the number of SF inspection repetitions. As in Figure 3, the false SF selection decreases as the number of SF inspection repetitions increases; the repetitions help to select the corresponding SF. However, many SF inspection repetitions increase the CAD time required by the receiver, which increases the transmitter’s preamble length.

When the range of the transmitter is between SF7 and SF9, inclusively, the probability of false SF selection is lower than when the range of the transmitter is between SF10 and SF12, inclusively; a lower SF has less quasi-orthogonality [13]. Thus, when more than three SF inspection repetitions occur at the receiver, the probability of selecting the wrong SF becomes very low (Equation (8)). However, in the range of SF10 to SF12, the receiver is more likely to detect the preamble of adjacent SFs; hence, the SF inspection repetitions cannot always guarantee a low probability of false SF selection. 

**Figure 4:**
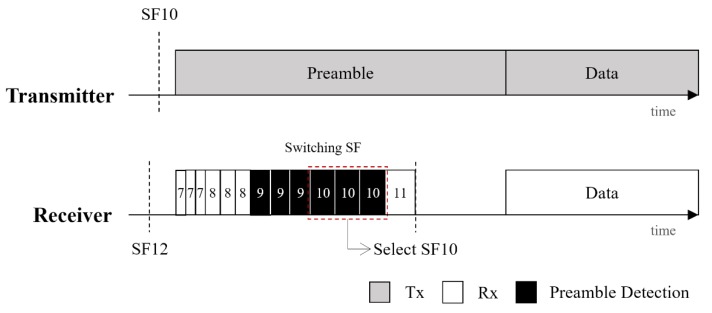
An example of the SF selection algorithm.

Considering these characteristics, we propose a modified SF selection algorithm at the receiver. In CAD mode, the receiver repeats the preamble inspection three times (*n* = 3) in ascending order from SF7. If the preamble is detected all three times in a specific SF, the receiver regards it as a candidate SF. If the candidate is SF7 or SF 8, the receiver immediately stops the SF inspection and chooses the corresponding SF; for SF7 and SF8, the probability of incorrectly selecting an adjacent SF is close to 0% where *n* is 3. For FS9, it is unlikely that the preamble will be detected three times by SF8 and SF10 of the receiver, but the preamble transmitted to SF10 may be detected three times by SF9 in 3.74% of the cases. Therefore, in the range from SF9 to SF12, the receiver continues to inspect the next SF until the preamble is not detected. Figure 4 shows an example of the modified SF selection algorithm when the transmitter sends a packet with SF10; the receiver continues to inspect the preamble in order from SF7 to SF11 even if it detects SF9 and SF10 three times. Because the preamble is not detected in SF11, the receiver stops the preamble inspection and selects SF10 to receive data. Through these two solutions, we can reduce false SF selection to 0%.

## 5. Performance Evaluation

In this section, we evaluate the performance of the ASFS scheme in single-hop and multi-hop LoRa networks.

### 5.1. Experimental Setup

To construct LoRa networks, we use two different modems: SX1272 and SX1301. With an 860 to 1020 MHz frequency range, both modems support 20 dBm output power as a maximum. We set the output power to 7 dBm for the experiments in a limited area. The SX1272 supports a single IF8 LoRa channel with a configurable bandwidth: 125 kHz, 250 kHz, or 500 kHz. In the IF8 LoRa channel, the transmitter and receiver communicate at a single data rate. The SX1301 is a high-performance transmitter and receiver. It supports both a single IF8 LoRa channel and multiple IF0 to IF7 LoRa channels. The IF8 LoRa channel is compatible with the SX1272 but does not support multi-data rates. The IF0 to IF7 LoRa channels only support a single bandwidth: 125 kHz. The SX1301 can simultaneously scan eight channels (IF0 to IF7) and demodulate up to eight packets with various data rates. A packet size for the experiment is fixed to 100 bits including physical layer (PHY) header and cyclic redundancy check (CRC).

**Figure 5:**
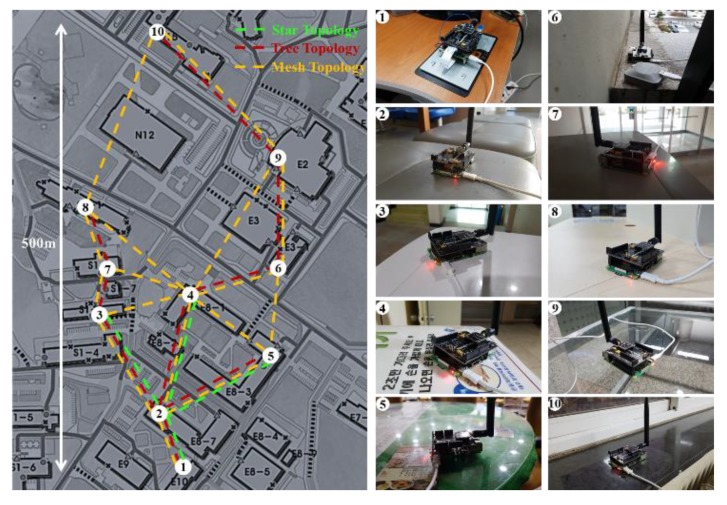
Experimental environments.

The LoRa modems connected to Raspberry Pi are installed at Chungbuk National University (Figure 5). A star topology connects five modems, and a tree topology and a mesh topology connect 10 modems each. Dotted lines between devices represent the communication links, and the SF used for each link is preset to the smallest possible value for communication. The star topology uses the existing LoRaWAN and the tree topology uses a simple packet forwarding method. We also implement a flooding algorithm used in [6] on the network layer for the mesh topology. The library used for the software implementation is as in [15]. We implemented the ASFS, tree topology, and mesh topology using the library on the SX1272.

### 5.2. Evaluation Result

To receive the packet correctly, the receiver must synchronize with the SF used by the transmitter. The SX1301 modem can demodulate packets sent in any SFs. However, because the SX1272 can only receive packets for a given SF, the transmitter and receiver must synchronize the SF before communication begins; this eventually restricts all network devices to one fixed SF for transmitting and receiving. For this reason, all devices must communicate with the worst SF and lowest data rate in the network. 

In the ASFS scheme, the receiver detects the preamble on all packets and sets the SF adaptively; it overcomes the constraint that all devices in the network correspond with one SF. For each network topology, Table 2 shows the results for the average SF and data rate of every link. When the SX1272 router is used, the SF of all devices must use the worst SF. In the mesh network, the link between devices 9 and 10 and the link between devices 4 and 9 can only communicate with SF12; thus, as all devices use SF12, this results in a lower average network data rate. Because the SX1301 module can receive any SF, each link independently defines the optimum SF. However, due to the difference in the maximum available bandwidth, with a lower average SF, the SX1301 router shows a lower data rate than the SX1272 router in the star and tree topology. When the SX1272 router uses the ASFS scheme (*n* = 3), it independently applies the optimal SF to each link like the SX1301 and uses a maximum bandwidth of 500 kHz. This results in a lower average SF and a higher average data rate for the network links.

**Figure 6:**
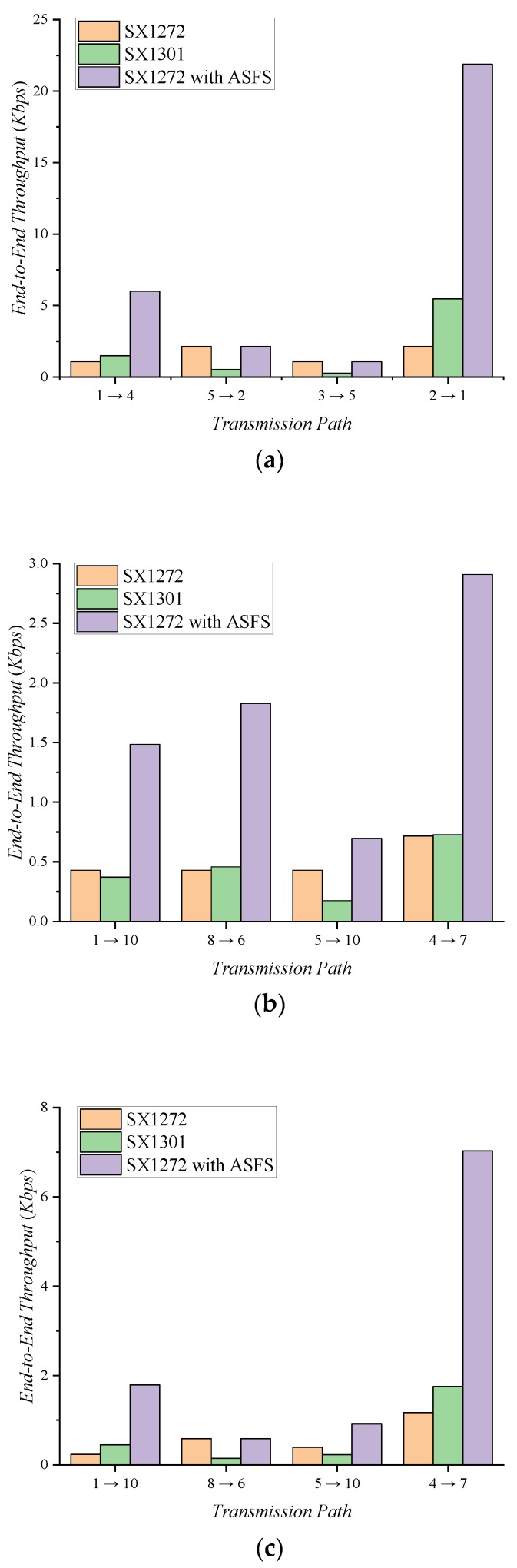
End-to-end throughput according to router type: (**a**) Star Topology; (**b**) Tree Topology; (**c**) Mesh Topology.

Figure 6 shows the maximum end-to-end throughput in each network topology; we assume traffic is only generated by devices included in the transmission path. The throughput of the SX1272 module depends on the data rate of each link and the hop count. In the mesh network, flooding causes packet collisions because all devices use the same channel and SF; this degrades end-to-end throughput. The flooding algorithm repeatedly broadcasts the packets until they reach the destination. To successfully communicate, each device must broadcast a packet at the highest SF of the connected links. Although the SX1301 can use an independent SF for each link, the flooding algorithm increases the average SF. The mesh topology also shows throughput limitations due to low bandwidth usage. In the star topology with SX1301 modules, a transmission path from devices 2 to 1 shows a throughput gain due to the low SF, but transmission paths from devices 5 to 2 and from devices 3 to 5 show much lower throughput than the SX1272 because there is no SF gain. In the tree topology, the SX1301 shows lower throughput compared with the others. 

SX1272 with ASFS supports high end-to-end throughput due to low average SF and high bandwidth per link. In the multi-hop network, it provides higher throughput than SX1272 and SX1301 due to lower SF of the path and higher bandwidth, respectively. Through the ASFS scheme, throughput is projected to substantially increase, and the network construction cost is reduced.

The star topology provides higher end-to-end throughput than the multi-hop topology, but the communication range is limited. In the multi-hop LoRa network, we found no suitable topology because the network performance differed based on the transmission path. In the future, we will study the network protocol for the multi-hop LoRa network using a different data rate per link.

To evaluate the network throughput, we performed an experiment using the star topology of Figure 5 with a gateway and three devices. We assume that three devices use independent SFs: SF7, SF9, and SF11, and all devices fairly generate traffic. For SX1272, the gateway sets one of SF7, SF9, and SF11 and receives a packet from a device. The SX1301 gateway uses multi-spreading factors with 125 kHz bandwidth. The SX1272 gateway with ASFS adaptively configures its SF using the ASFS scheme.

**Figure 7:**
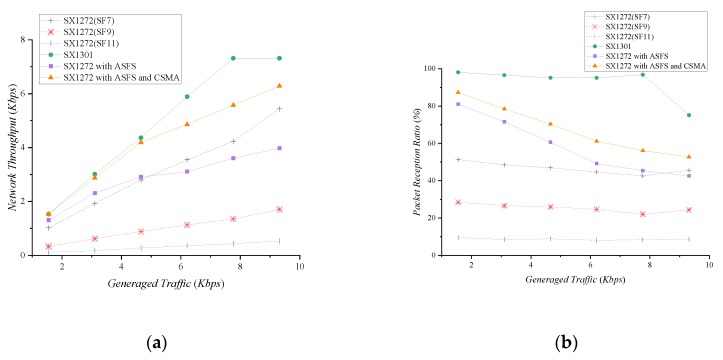
Network throughput and packet reception ratio with respect to network traffic volume: (**a**) Network Throughput; (**b**) Packet Reception Ratio.

Figure 7 shows the network throughput and packet reception ratio with respect to the generated traffic in the network. The experimental results are the average of 10 data collected over 100 seconds. The network throughput increases as the traffic generated by the network grows. SX1272 shows the highest throughput in SF7, which provides higher data rate than other SFs. However, since the SX1272 gateway cannot receive the packets transmitted by the SF which is not set, the packet reception ratio (PRR) is low as seen in Figure 7b. The SX1301 can receive eight packets simultaneously for all SFs. It shows a much higher throughput and packet reception ratio than the SX1272. The low bandwidth usage of SX1301 makes the channel more saturated than the SX1272. As shown in Figure 7, the throughput and PRR become saturated when the traffic generated by the network is 7.76 Kbps. Nevertheless, the SX1301 still can use additional SFs or channels to further increase a network throughput. This has a great advantage in accommodating multiple devices in the network.

If each device transmits packets in different SFs, no packet collisions occur, but the SX1272 gateway can only receive one packet at a time. This feature allows ASFS to receive a packet sent from the SF of the preamble sampled first. If there is little traffic on the network, it is unlikely that packets will be transmitted at the same time; hence it represents relatively high throughput and PRR. However, as generated traffic in the network increases, the probability of transmitting packets at the same time decreases the throughput and PRR. Although packet collisions do not occur, packets transmitted with different SFs are ignored by the receiver. To reduce this effect, we use carrier sense multiple access (CSMA) [16], a representative medium access control mechanism. By reducing the probability that the devices transmit packets simultaneously through carrier sensing, it shows higher throughput and PRR than when using ASFS alone. When the generated traffic in the network is low, it shows almost the same throughput as the SX1301.

## 6. Conclusions

In this paper, we proposed an ASFS scheme to enhance throughput by supporting multi-spreading factors with inexpensive single-channel LoRa modules. It can be implemented with two simple methods: iterative SF inspection and a modified SF selection algorithm. These simple methods overcome hardware constraints without affecting existing LoRa behavior. We evaluated the performance of the ASFS scheme by implementing an existing single-hop network and a multi-hop network configuration that was not provided by LoRaWAN. The ASFS scheme showed outstanding end-to-end throughput performance, but the SX1301 showed even better results. Nevertheless, an improved algorithm could further increase throughput for the SX1272 with ASFS. We expect the ASFS scheme will be a suitable technology for applications requiring high throughput on a multi-hop network.

## Figures and Tables

**Table 1:** *T_PREAMBLE_* and cumulative *T_CAD_* according to SFs. The unit is *ms*.

	SF7	SF8	SF9	SF10	SF11	SF12
*T_preamble_*	2.624	5.248	10.496	20.992	41.984	83.968
cumulative *T_CAD_*	0.32	0.896	1.984	4.096	8.256	16.152

**Table 2:** Average performance comparison of network links.

Network Topology	Router Type	Spreading Factor	Data Rate (Kbps)
**Star**	SX1272	11	2.148
	SX1301	10	1.77
	SX1272 with ASFS	10	7.080
**Tree**	SX1272	11	2.148
	SX1301	9.44	2.105
	SX1272 with ASFS	9.44	8.420
**Mesh**	SX1272	12	1.172
	SX1301	9.75	1.730
	SX1272 with ASFS	9.75	6.921
